# Towards better antivenoms: navigating the road to new types of snakebite envenoming therapies

**DOI:** 10.1590/1678-9199-JVATITD-2023-0057

**Published:** 2023-12-18

**Authors:** Suthimon Thumtecho, Nick J. Burlet, Anne Ljungars, Andreas H. Laustsen

**Affiliations:** 1 Division of Toxicology, Department of Medicine, Chulalongkorn University, King Chulalongkorn Memorial Hospital, the Thai Red Cross Society, Bangkok, Thailand.; 2 Department of Biotechnology and Biomedicine, Technical University of Denmark, Kongens Lyngby, Denmark.

**Keywords:** Antivenom, Next-generation antivenom, Recombinant antivenom, Antivenom development, Snakebite envenoming, Small molecule toxin inhibitors, Toxin-neutralization

## Abstract

Snakebite envenoming is a significant global health challenge, and for over a century, traditional plasma-derived antivenoms from hyperimmunized animals have been the primary treatment against this infliction. However, these antivenoms have several inherent limitations, including the risk of causing adverse reactions when administered to patients, batch-to-batch variation, and high production costs. To address these issues and improve treatment outcomes, the development of new types of antivenoms is crucial. During this development, key aspects such as improved clinical efficacy, enhanced safety profiles, and greater affordability should be in focus. To achieve these goals, modern biotechnological methods can be applied to the discovery and development of therapeutic agents that can neutralize medically important toxins from multiple snake species. This review highlights some of these agents, including monoclonal antibodies, nanobodies, and selected small molecules, that can achieve broad toxin neutralization, have favorable safety profiles, and can be produced on a large scale with standardized manufacturing processes. Considering the inherent strengths and limitations related to the pharmacokinetics of these different agents, a combination of them might be beneficial in the development of new types of antivenom products with improved therapeutic properties. While the implementation of new therapies requires time, it is foreseeable that the application of biotechnological advancements represents a promising trajectory toward the development of improved therapies for snakebite envenoming. As research and development continue to advance, these new products could emerge as the mainstay treatment in the future.

## Background

Snakebite envenoming represents a persistent and significant global health challenge, with an annual global incidence estimated to be between 1.8 - 2.7 million envenomings, leading to approximately 81,000 - 138,000 fatalities and many more indefinable debilitating consequences [1-3]. Despite the longstanding history of human exposure to snakebite envenomings [2], antivenoms consisting of plasma-derived antibodies (or fragments thereof) from hyperimmunized animals have been the primary treatment option since they were first developed by Albert Calmette, Césaire Phisalix, and Gabriel Bertrand in the late 19^th^ century [4, 5]. The proven efficacy of these antivenoms in preventing fatalities [6] has led to their recognition as essential medicines by the World Health Organization (WHO) [7]. Consequently, antivenoms have been integrated into standard treatment guidelines for snakebite envenoming that are implemented worldwide [8], resulting in serum institutes having set up large-scale production of these products in many countries. Nevertheless, traditional antivenoms come with several drawbacks related to their heterologous nature and production method. In particular, the non-human proteins present in antivenoms (including the antibodies themselves) may trigger immunogenic reactions in snakebite victims, such as serum sickness or anaphylaxis, and many antivenom products suffer from batch-to-batch variation and a relatively low content (and/or unbalanced composition) of therapeutically relevant antibodies [9, 10]. Moreover, although paraspecificity (binding to structural similar antigens/toxins that were not included in the immunization mixture) can occur, antivenoms are typically mostly effective against the venom(s) from the snake species that they have been raised against [11, 12]. Finally, the laborious and low-throughput manufacturing process used to produce antivenoms results in several issues. These include a risk of incorporating impurities of animal origin and the potential for vertical transmission of diseases even after the purification process. Additionally, the relatively high cost of goods that antivenoms have may impose a significant economic burden on snakebite victims and/or healthcare systems in low-income areas with a high incidence of snakebite envenoming [13]. This latter aspect, in particular, is critical for the deployment of antivenoms, as most snakebite victims worldwide are found amongst poor, rural populations [3, 14].

While the clinical management of snakebite envenoming is multifaceted and involves not only medical intervention but also logistics, training, diagnostics, and economic considerations, antivenoms that can neutralize snake venoms remain a cornerstone of modern envenoming therapy [15, 16]. Given the persistent obstacles of traditional antivenoms, there is a need to develop new types of antivenom products that are safer, more effective, and affordable [2, 17]. While antivenom researchers and manufacturers have made strides in optimizing product efficacy, safety, stability, and neutralization capacity across species [18-20], new technological advances now offer an opportunity for rethinking how antivenom products could ideally be developed and manufactured to improve this important type of medicines. In this review, we discuss key aspects that should be considered before engaging in the development of new types of antivenom products, present various molecular formats that might be utilized in these, and mention recent technological advancements that have the potential to improve the development process. Other clinically important aspects surrounding optimal envenoming therapy, including timely and correct administration of appropriate doses, treatment of secondary effects and symptoms, and optimal care and nursing of patients, can be found elsewhere [3, 16]. 

### Requirements for new types of antivenom products 

**Figure 1.**
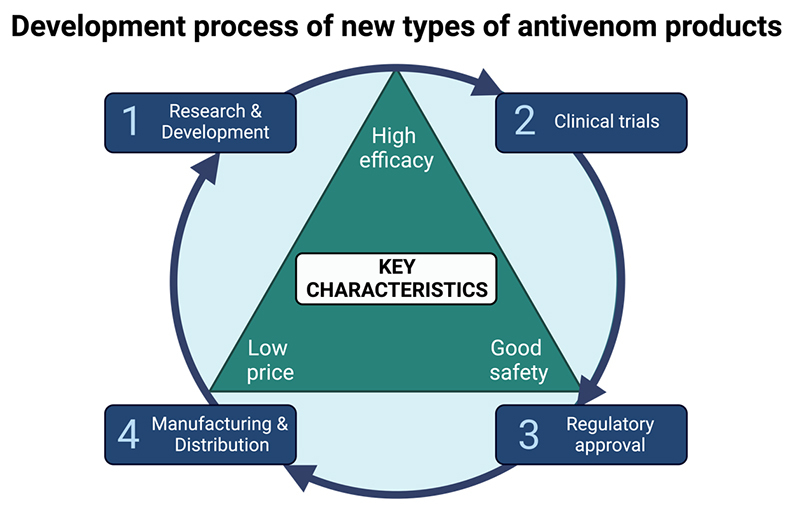
Key considerations for antivenom development. The development of new types of antivenom products requires careful consideration of key characteristics, including high efficacy, favorable safety profiles, and affordability. These critical aspects should be addressed throughout the product development process, starting from conception and extending to manufacturing. Comprehensive clinical assessments and rigorous approval processes are indispensable to ensure the efficacy and safety of antivenom products. Simultaneously, cost-effectiveness plays a pivotal role in ensuring the accessibility of antivenoms in regions where they are needed. It is thus essential to have the end product in mind when developing new types of antivenom products, as well as to have a holistic overview of the process. The figure was created with BioRender.com.

When developing new types of antivenom products, a comprehensive approach spanning from bench to bedside needs to be taken (Figure 1). First and foremost, new types of antivenoms need to demonstrate improved clinical efficacy compared to traditional antivenoms [9, 19]. The efficacy of traditional antivenoms relies on the antibodies generated through the immune response of the production animals against the toxins in the venom(s) used for their immunization [21-23]. However, venoms are complex mixtures containing multiple toxins with varying functions, abundance, toxicity, size, and immunogenicity [19, 24]. As a result, not all medically important toxins in the venom(s) elicit a sufficiently strong immune response in the animals to trigger the production of neutralizing antibodies, consequently limiting the neutralizing capacity of the resulting antivenom(s) against some toxins [25-28]. Therefore, a thorough understanding of snake venom complexity is necessary to help guide the development of an antivenom product that can eliminate the venom from the patient’s body and ideally neutralize all medically relevant toxins [24]. To this end, particularly toxicovenomics, which combines venomics and toxicity studies of individual venom components, can be used to identify the medically most important toxins that require neutralization [29-31]. Having this knowledge provides the opportunity to isolate or recombinantly express and use these toxins as targets in rational drug discovery campaigns to find toxin-neutralizing agents that can be used to formulate new types of antivenom products [32-35]. 

In addition to being more efficacious in terms of neutralizing capacity, new types of antivenom products should preferably be polyvalent, *i.e.*, be able to neutralize the venoms of multiple snake species, similar to many traditional antivenoms [36]. To minimize the required number of toxin-neutralizing agents needed in an antivenom product while still effectively neutralizing all medically important toxins of several snake species, a carefully designed mixture of broadly neutralizing agents targeting multiple (similar) toxins can be utilized [37-43]. Within antibody research, one strategy to discover such broadly neutralizing agents against multiple toxins involves the use of phage display technology [42-45]. This technology allows antibody fragments to be discovered *in vitro* against, in principle, any target toxin regardless of its immunogenicity and toxicity [43, 46-48] and can be set up in a way that facilitates the discovery of cross-reactive antibodies capable of binding multiple similar toxins [42, 43, 45]. Another approach for the discovery of cross-reactive neutralizing agents involves the use of recombinantly produced consensus toxins. These artificial toxins are designed to resemble an average sequence of several related toxins and can be used for immunization [49, 50] or as target antigens during phage display-based discovery campaigns [49, 51]. Lastly, cross-reactive, high-affinity neutralizing agents can be discovered or optimized by applying recent advances within the field of artificial intelligence, next-generation sequencing, and machine learning [52, 53]. The combination of these techniques presents an opportunity to either design novel high-affinity neutralizing agents or improve existing neutralizing agents both in terms of affinity and/or cross-reactivity *in silico* [54], which could help speed up and improve the development process for new types of antivenoms. 

Beyond the neutralization of all medically relevant toxins, the clinical efficacy of antivenom products is further influenced by the interplay between their pharmacokinetic properties (which include how they are administered, distributed, metabolized, and eventually eliminated from the body) and the toxicokinetics of venoms [55]. The toxicokinetics of whole venoms are typically complex due to the presence of multiple toxins with varying kinetic properties, which can include fast systemic distribution, rapid deep tissue penetration, slow elimination from the body [56], and the gradual release of toxins from the bite site and into circulation over time (known as the depot effect) [57, 58]. While the use of smaller therapeutic agents might facilitate rapid tissue penetration and organ access, larger molecules tend to remain in circulation for longer periods [59]. Therefore, when selecting the most optimal format(s) for the therapeutic agents in an antivenom product, it is important to consider their inherent pharmacokinetic properties to ensure that these fit the toxicokinetics of the target venom(s). This entails that toxins must be neutralized before their toxic effects become too detrimental to the victim and that the antivenom has a sufficient duration of action to neutralize toxins that may leave the bite site and enter circulation a long time after the bite occurred. To achieve this, a combination of different therapeutic formats in a single antivenom product may be necessary, as no single therapeutic agent is superior across all properties [55].

Alongside efficacy, a good safety profile is essential for new types of antivenom products. Today, due to the risk of severe adverse reactions, traditional antivenoms are typically administered only after the onset of clinical manifestations [60]. This delay in administration allows the injected toxins to further exert their toxic effects, which can lead to patient distress, prolonged hospital stays, and irreversible complications, such as tissue necrosis [61, 62]. Therefore, to achieve timely intervention and mitigate the risk of adverse reactions, the products must be free from contaminations, possess very low immunogenicity, and show no off-target effects, which refer to unintended and undesired interactions between the therapeutic agents in the antivenom and non-target molecules (such as host proteins). To improve the safety profiles, removing the reliance on animal-derived components should be a priority for the development of new types of antivenoms. For this, an opportunity may lie in the utilization of recombinant expression systems (bacteria, yeast, and mammalian cells) to produce protein-based binding molecules [63, 64], or in the chemical synthesis of, for example, small molecule inhibitors [3].

To evaluate the efficacy and safety of antivenoms, a comprehensive preclinical and clinical assessment is essential. For preclinical assessment, the current recommendation by the WHO is to evaluate the ability of antivenoms to neutralize venom-induced lethality in rodent models that involve pre-incubation of venom and antivenom for 30 minutes before being injected [65]. To more closely mimic a real-life snakebite scenario, rescue assays, where the animal is exposed to the toxin or venom before administration of the antivenom, can be conducted. While it is more challenging to perform such assays in a standardized manner, rescue assays enable a more thorough assessment of the therapeutic utility of an antivenom product, including its efficacy, pharmacokinetics, and pharmacodynamics, as well as providing a better insight into the toxicokinetics of the venom [66, 67]. Moreover, conducting supplementary *in vitro* assays to evaluate the neutralization of venom-induced pathologies, such as hematotoxicity, cytotoxicity, and neurotoxicity, allows for a more comprehensive preclinical assessment beyond lethality alone and can be used to reduce the number of animals needed for experiments [65, 68-72]. 

After a thorough preclinical evaluation, new antivenom products should undergo a well-designed clinical assessment. Surprisingly, this has not been done for many traditional antivenoms [73] since clinical trials are not mandatory to obtain approval for plasma-derived antivenoms and their use in clinical settings [28]. Ideally, clinical trials for new (and traditional) types of antivenom products should be prospective, comparative, interventional, and well-conducted to confirm safety and efficacy in humans [73-75]. Beyond the design of the clinical trials themselves, a swift and efficient approval process for all types of antivenom products would be beneficial, as this could facilitate timely access to more effective and safer therapeutic alternatives for patients. To accelerate the approval processes, common guidelines between different national regulatory agencies and simplified regulatory pathways, such as a shortened route for biological products, could be implemented [76]. Another approach to expedite the approval process could involve repurposing drugs that have already undergone clinical trials for other indications than snakebite envenoming to potentially bypass the need for some of the early clinical trials (such as phase 1 trials on healthy volunteers) [77]. 

Lastly, when developing new types of antivenom products, it is important to estimate their final market price, considering that affordability is a key factor for therapies that are to be deployed in low-income regions [13, 78]. To ultimately reduce overall costs related to both development and manufacturing, the development of new types of antivenom products should employ versatile and standardized approaches during the discovery process as well as scalable production technologies during the manufacturing [79]. For example, by applying *in vitro* display technologies for the discovery of recombinant antibodies or antibody fragments and by employing microbial or mammalian expression systems for large-scale production, an antivenom with lower costs than traditional antivenoms could potentially be manufactured [40, 80]. Alternatively, development costs, especially those related to the discovery of new toxin-neutralizing molecules and running clinical trials, can be reduced by utilizing repurposed medications with established pharmacokinetic properties and safety profiles [81, 82]. Moreover, expanding the market by developing polyvalent antivenom products that can be used across larger regions and against multiple snake species could potentially enable manufacturers to achieve higher production volumes and better economies of scale [13, 83, 84]. Consequently, this may help make antivenom products more affordable for victims and healthcare systems [85], addressing the importance of affordability in regions with limited resources.

### Relevant toxin-neutralizing agents for new types of antivenom products

**Figure 2.**
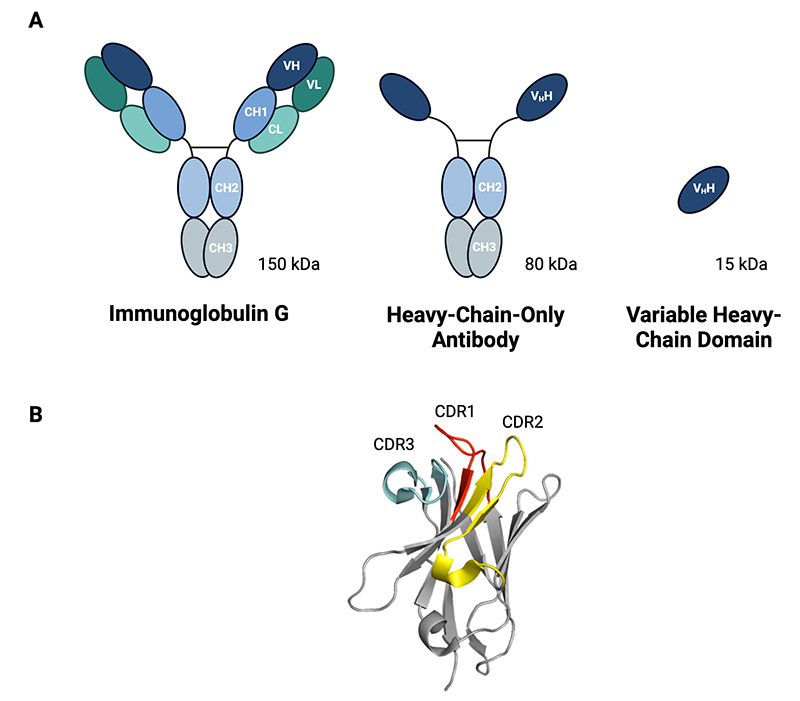
Different antibody formats that have been investigated for their utility in neutralizing snake toxins. **(A)** Schematic representations of an immunoglobulin G (IgG) antibody, a heavy-chain-only antibody (HCAb), and a variable heavy-chain domain (V_H_H). IgG antibodies are composed of four polypeptide chains: two identical heavy chains (H) and two identical light chains (L), forming a flexible Y-shaped structure. Each chain contains a variable (V) region and one or more constant (C) region(s). In contrast, camelids produce a unique type of antibodies, consisting of only heavy chains known as HCAbs. When expressed alone, the variable domains of HCAbs are referred to as V_H_Hs and are notably smaller in size compared to the IgG. **(B)** The crystal structure of a representative V_H_H (PDB ID 4PPT). The complementarity-determining regions (CDRs), particularly the long flexible loop of CDR3, are highly variable and crucial for antigen binding. CDRs 1, 2, and 3 are indicated in red, yellow, and blue, respectively. The figure was created with BioRender.com.

**Figure 3.**
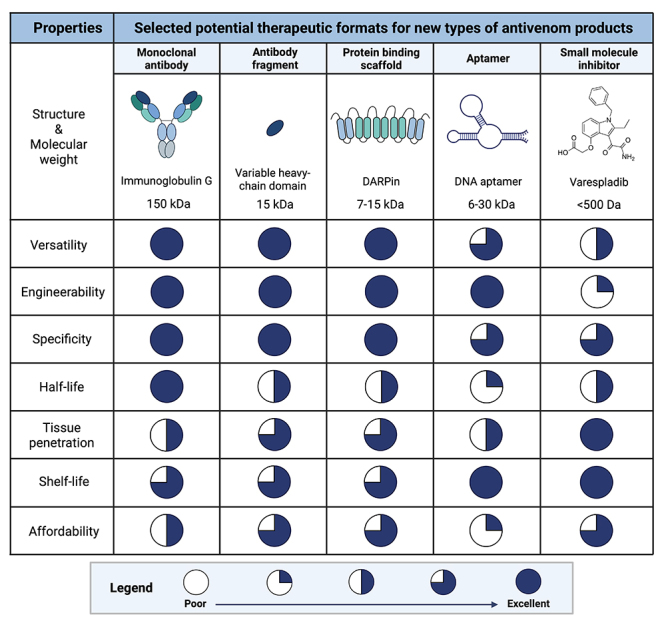
Characteristics of selected therapeutic agents that could potentially be used for the development of new types of antivenom products, including their structures, molecular weights, and important therapeutic properties. The Harvey balls visually illustrate the theoretical favorability of each property, represented by the dark blue area, and based on the professional, yet arguably subjective, expert evaluation by the authors. It is essential to acknowledge that the Harvey balls are meant to capture the general characteristics of each type of molecular scaffold/class and may not apply to specific molecular agents within each class, which could have properties that deviate from the mean. Versatility indicates the ability to neutralize different types of toxins, while engineerability refers to the ease of modifying molecules from the class to achieve specific properties or functions, such as half-life extension. Specificity refers to the ability to selectively interact with a specific target, thereby minimizing off-target effects (unintended interactions that could lead to undesired effects). Half-life represents the time it takes for half of the administered therapeutic agent to be cleared from the body. Tissue penetration denotes the capability of effectively reaching and binding to target toxins in tissues, including deep tissues and various organs. Shelf-life indicates how long an agent maintains its activity under cool storage conditions. Affordability highlights the cost-effectiveness of the therapeutic agent. Safety is deliberately not included, although it is an essential therapeutic property, as this should be evaluated on a case-by-case basis for each specific molecule in a class. The figures were created with BioRender.com, and the structure of Varespladib corresponds to PubChem CID 155815. The size of the molecules is not drawn to scale.

Various therapeutic agents can be considered for the development of effective, safe, and affordable antivenom products, each with their own set of advantages and disadvantages (Figure 2). While polyclonal antibodies, including immunoglobulin G (IgG) antibodies and their fragments, are common therapeutic formats used in traditional antivenoms [4, 5], the conventional method of immunizing animals with whole venom(s) presents challenges in generating antibodies against some specific toxins and results in end products with low therapeutic contents [9, 10]. Consequently, there is an interest in more specific therapies based on common types of antibodies, such as monoclonal IgG antibodies and antibody fragments, which are well-validated classes of therapeutic agents already used for multiple indications, such as autoimmune diseases, cancers, and infections [86-90]. Besides their proven efficacy as therapeutics, recombinantly produced monoclonal IgG antibodies have also been in focus in snakebite envenoming research due to their good safety profiles, long half-lives in circulation (typically around three weeks) [91], and the growing evidence of their effectiveness in neutralizing both specific toxins and whole venom-induced lethality *in vivo* [38, 43, 92]. By utilizing strategies for the discovery of broadly neutralizing antibodies and combining such antibodies in carefully designed oligoclonal mixtures, an antivenom targeting multiple similar and dissimilar toxins found in the venoms of various snake species can likely be achieved [42-44]. In addition to monoclonal IgG antibodies, which consist of two light-chains and two heavy-chains (Figure 3), smaller antibody fragments comprised of only one domain, called single-domain antibodies (sdAbs), are being investigated for potential use as toxin-neutralizing agents. Among these, there is a particular focus on variable heavy-chain domains (V_H_Hs or nanobodies) [46], which are variable domains derived from heavy-chain-only antibodies naturally found in camelids. V_H_Hs can have comparable toxin-neutralization capacities to IgGs [93] and possess a long flexible loop in their antigen-binding site, which allows them to bind to buried epitopes within protein structures that are typically inaccessible to antibodies [94]. The much smaller size of V_H_Hs (and sdAbs in general) compared to IgGs (15 kDa versus 150 kDa) might enable these smaller fragments to penetrate deep tissue faster than IgGs [95] (Figure 3). Furthermore, V_H_Hs offer the advantages of high solubility and high thermal stability, withstanding temperatures of 60-80 °C, and often have the potential to refold into an active form upon thermally induced denaturation [95, 96]. 

Monoclonal antibody-based therapeutics will likely offer improved safety profiles compared to plasma-derived antibodies as they enable the formulation of an end product with a high therapeutic content, thus allowing for the administration of much lower doses compared to traditional antivenoms. In addition, monoclonal antibodies can be designed to be more compatible with the human immune system than heterologous polyclonal antibodies. This can be achieved by developing them as fully human antibodies**,** where the entire molecule is of human origin, or as humanized antibodies, where the non-human complementarity-determining regions in the variable domains responsible for antigen binding are incorporated into a human antibody framework. Depending on their animal origin, non-human antibodies or antibody fragments can also possess low immunogenicity even without any engineering, as is the case with V_H_Hs from camelids. The typical low immunogenicity of V_H_Hs partially arises from their much smaller size compared to IgGs, resulting in a lower amount of protein needed for neutralization [97], as well as their high solubility (that makes them less prone to aggregation). Additionally, their high sequence homology to human antibodies often makes humanization unnecessary [98, 99]. To obtain fully human antibodies, *in vitro* discovery strategies, such as phage display technology [100], or *in vivo* discovery strategies, including the use of transgenic animals, such as transgenic mice that have been genetically engineered to produce human antibodies, can be used [101]. 

Monoclonal antibodies can be further engineered to have specific pharmacokinetic properties. One example involves the extension of the half-life of IgGs in circulation through mutations in the crystallizable fragment (Fc) to increase their affinity to the neonatal Fc receptor (FcRn), which protects the antibodies from lysosomal degradation and enhances their recycling properties [102]. Likewise, the binding between the Fc domain and FcRn can be exploited to prolong the inherently short half-life of V_H_Hs in circulation (about 1.5 hours) [103] by fusing the V_H_Hs with a human Fc domain or by making a bivalent construct combining a toxin-binding V_H_H with an albumin binding V_H_H, as albumin also binds to FcRn [95, 104-106]. In addition, the half-life of V_H_Hs can be extended by increasing the molecular weight above the renal filtration threshold, for example, by assembling them into multimers [107] or by fusing them with larger molecules that have long half-lives, such as albumin [108-110]. 

To keep the production cost of monoclonal antibodies low, established and validated large-scale manufacturing setups of widely used monoclonal antibody-based therapeutics can be utilized [111]. Moreover, employing antibody fragments with simple structures, such as sdAbs, can likely further reduce the production cost, as these can be expressed using microbial expression systems, which upon optimization can reach very high titers and high productivity during fermentation [112-115]. Finally, the overall cost of the final antivenom products could potentially be reduced by implementing more stable formats to eliminate the expenses associated with temperature-controlled supply chains during product distribution [116].

Apart from antibodies and fragments thereof, non-antibody-based therapeutic agents are being considered by some research groups for the development of new types of antivenom products [117]. Examples of such molecules are protein binding scaffolds, which are synthetic peptide frameworks, and aptamers, which are short single-stranded oligonucleotides [118, 119]. Similar to antibodies, these molecules allow for the design and discovery of constructs that have high affinity and specificity to a wide spectrum of targets, including toxic and non-immunogenic ones [120, 121]. As an example, recent studies have shown that aptamers can be designed to neutralize toxins from some important snake toxin families, such as phospholipases A_2_ (PLA_2_s), neurotoxins, and cytotoxins [122-124]. Similarly, it has been demonstrated that designed ankyrin repeat proteins (DARPins), a specific type of protein binding scaffold that mimics and enhances the functionality of natural ankyrin repeat proteins involved in protein-protein interactions [125], can be used to neutralize toxins [125-127]. This suggests that they can be used for the development of antivenom products [119, 125]. In addition to their demonstrated capabilities of being able to neutralize selected toxins, these non-antibody-based molecules share several similar advantageous characteristics with V_H_Hs, including their high thermal stability [119, 128, 129] and small sizes (7 to 15 kDa for protein binding scaffolds and 6 to 30 kDa for aptamers), which could allow for rapid tissue penetration [119, 121, 130]. Furthermore, both molecules can be manufactured on a large scale [129, 131, 132], although the actual cost of producing large quantities of aptamers remains elusive, and we as authors are skeptical about whether this is on par with the cost of manufacture of antibodies and other proteins [9]. Typically, aptamers can be chemically synthesized from a library of different nucleic acid sequences. Protein scaffolds, such as DARPins, generally do not need mammalian cells for production and can be efficiently expressed in bacterial or fungal expression systems due to the absence of disulfide bonds and lack of required post-translational modifications in these molecules [40, 132]. Regarding safety, clinical data from a few U.S. Food and Drug Administration (FDA)-approved products based on protein binding scaffolds [131, 133-135] and one aptamer product [136, 137] indicate that these types of scaffolds can have a relatively good safety profile and show low immunogenicity, although their general safety remains inconclusive and necessitates further clinical data [121, 138]. Similar to other low molecular weight molecules, both protein binding scaffolds and aptamers carry the drawback of a short circulatory half-life [139]. In addition to this, unmodified aptamers are unstable under physiological conditions due to their general susceptibility to hydrolysis by ubiquitous nucleases in human serum [120], which can quickly shorten their half-life, sometimes even down to the orders of seconds for single-stranded hydroxy nucleic acid or ribonucleic acid aptamers [140]. To extend the half-life of these two types of therapeutic agents, strategies that increase their sizes, such as conjugating them with polyethylene glycol (PEG) or albumin, similar to sdAbs, can be employed [131, 141-146]. However, it is important to consider that modifications made to these molecules may significantly affect their overall manufacturing complexity and cost. This concern is particularly pronounced for aptamers, as the application of such half-life extension technologies would necessitate additional steps in the manufacturing process, including chemical synthesis/conjugation and/or recombinant expression. In some instances, the use of such half-life extension technologies can also compromise the efficacy, pharmacokinetics, and safety profiles of the therapeutic agents [142, 145, 147, 148]. While protein binding scaffolds could potentially find utility in snakebite envenoming therapies, we find it challenging to envision how their benefits exceed those of antibody-based therapeutic agents [101, 149], although we acknowledge that, with improved *in vitro* discovery approaches, it may one day be possible that protein binding scaffolds become a competitive alternative [150]. In contrast, it seems highly speculative that aptamers will find utility as therapeutics in clinical snakebite management, given the inherent developability liabilities and the number of therapeutic drawbacks of this molecular scaffold [140].

Another therapeutic modality to consider for the development of new types of antivenom products is small molecule inhibitors [37]. These molecules typically function by binding to the active site of enzymatic toxins, which potentially enables them to inhibit an entire class or family of toxins that share similar active sites. In contrast to biological drugs, many small molecule inhibitors are typically not susceptible to enzymatic degradation in the gastrointestinal tract and can often be administered orally (sometimes as prodrugs) [81]. In some settings, this route of delivery can be beneficial since it enables easy administration (if the patient is conscious and not an infant) before hospital arrival, which may allow for an earlier start of treatment for patients living far away from a hospital or clinic. Early administration is particularly crucial when targeting toxins that rapidly distribute in circulation and penetrate deep tissues [77, 151] and toxins that cause irreversible damage [152]. Once administered, some small molecule inhibitors may rapidly distribute in the body and can reach the active sites of enzymes, including those that are typically difficult to access by larger molecules. Because small molecule inhibitors are produced through chemical synthesis and often can be manufactured in large quantities via cost-effective and validated methods, they could be a promising option for the development of more affordable antivenom products [153]. However, it is important to note that different small molecule inhibitors require different manufacturing processes [154], which might make production facilities less versatile compared to those used for the production of standard protein formats, such as antibodies. It is also crucial to highlight that repurposing small molecule inhibitors, which have already gone through clinical development, differs significantly from attempting to discover novel inhibitors. Small molecule drug discovery faces very high attrition rates, where drug candidates frequently fail to advance through subsequent stages of development [155] compared to, for example, standard antibody formats [79, 156].

To date, several of the small molecule inhibitors that have been evaluated for their ability to neutralize snake toxins are repurposed pharmaceuticals that were initially developed for other indications [157, 158]. For instance, Varespladib, originally developed for ulcerative colitis, rheumatoid arthritis, asthma, sepsis, and acute coronary syndrome [159], has been shown to inhibit the toxic effects of snake PLA_2_s [159, 160]. Similarly, snake venom metalloproteinase inhibitors initially developed for cancer therapy, such as batimastat and marimastat [161-165], and metal ion chelators, such as ethylenediaminetetraacetic acid (EDTA) and 2,3-dimercapto-1-propanesulfonic acid, have shown promise for being repurposed for treatment of snakebite envenoming [84, 166]. Although clinical efficacy data is pending, repurposing these or similar molecules that have already been proven safe in early clinical trials may reduce the development risk and timeline for therapeutic agents entering the clinic [77], as it may circumvent the need to perform clinical phase I safety studies and the need to initiate an entirely new resource-intensive discovery and development process [81]. 

Although several antibody and non-antibody-based therapeutic agents discussed in this review are still in the early stages of investigation and require further validation of both efficacy and safety, some of them have demonstrated promising results in preclinical studies, indicating their potential to be included in new types of antivenom products [59, 117, 119]. By combining various types of therapeutic agents that comprise different characteristics and target different venom toxins, along with knowledge about venom complexity and toxicokinetics, we believe that it may become feasible to develop new types of antivenom products that can neutralize (all) medically relevant toxins and are tailored to possess favorable pharmacokinetic profiles.

## Conclusion

To overcome some of the challenges associated with traditional antivenoms, there is a need to develop new types of antivenom products that possess better efficacy and safety while being affordable at the same time. To achieve this, modern biotechnological methods could be applied in the development of new therapeutic agents with different neutralizing capacities and pharmacokinetic profiles. By combining various therapeutic agents, it is possible to develop new types of antivenom products that are broadly neutralizing, safe, quality-assured, and cost-effective to produce on a large scale using standardized manufacturing platforms. So far, monoclonal antibodies and antibody fragments stand out as the most promising types of versatile molecular scaffolds that possess these attributes. Meanwhile, selected small molecule inhibitors repurposed from other drug development programs may find utility in some cases (against specific toxin families), as their established manufacture and former clinical assessment may fast-track them through the regulatory approval process. However, acknowledging the time-consuming undertaking of developing, evaluating, and obtaining regulatory approval for new antivenom products, traditional antivenoms will likely remain a therapeutic cornerstone within the treatment of snakebites for now. In the meantime, alternative therapeutic options might also see the light of day, such as fortifying existing antivenoms with, e.g., repurposed small molecule inhibitors, monoclonal antibodies, or fragments thereof. Nevertheless, the application of biotechnological innovations and close collaborations between researchers, engineers, clinicians, regulatory agencies, and funders across multiple countries could potentially deliver new types of envenoming therapies to snakebite victims worldwide. 

## Abbreviations

CDR: Complementarity-determining region; DARPin: Designed ankyrin repeat protein; EDTA: Ethylenediaminetetraacetic acid; Fc: Crystallizable fragment; FcRn: Neonatal Fc receptor; FDA: Food and Drug Administration; IgG: Immunoglobulin G; PLA_2_: Phospholipase A_2_; sdAb: Single-domain antibody; V_H_H: Variable heavy-chain domain; WHO: World Health Organization.
